# Coexistence of plasmablastic lymphoma and adenocarcinoma in the stomach: a case report and literature review

**DOI:** 10.1007/s13691-025-00751-4

**Published:** 2025-02-15

**Authors:** Takato Maeda, Takenori Takahata, Shintaro Goto, Takao Oyama, Satoru Nakagawa, Yasuhisa Murai, Ryuma Machida, Nao Ishidoya, Juichi Sakamoto, Hideki Iwamura, Hirotake Sakuraba

**Affiliations:** 1 Department of Gastroenterology, Tsugaru General Hospital, 12-3 Iwaki-Cho, , Goshogawara-Shi, Aomori 037-0074 Japan; 2 Department of Gastroenterology, Hematology and Clinical Immunology, Hirosaki University Graduate School of Medicine, 5 Zaifu-Cho,, Hirosaki-Shi, Aomori 036-8562 Japan; 3 Department of Pathology and Bioscience, Hirosaki University Graduate School of Medicine, 5 Zaifu-Cho, Hirosaki-Shi, Aomori 036-8562 Japan

**Keywords:** Plasmablastic lymphoma, Gastric adenocarcinoma, Stomach, Synchronous tumor, Case report

## Abstract

Plasmablastic lymphoma (PBL) is a rare B-cell lymphoma. Reports on primary gastric PBL are limited, and its endoscopic features remain poorly understood. We report a case of gastric PBL with multiple polypoid lesions in an immunocompetent individual. A 72-year-old man presented with upper abdominal discomfort. Esophagogastroduodenoscopy (EGD) revealed multiple raised lesions of variable sizes in the stomach, prompting a tumor biopsy. Based on histopathological findings, diffuse large B-cell lymphoma was suspected. Rituximab, cyclophosphamide, doxorubicin, vincristine, and prednisone (R-CHOP) chemotherapy was administered. After six cycles of R-CHOP therapy, EGD showed a partial reduction of the gastric tumor, and a biopsy was performed on the remaining tumor. Histopathology was re-examined, and immunohistochemical analysis revealed that the tumor cells were plasmablastic and strongly positive for both CD38 and CD138. The cells showed cytoplasmic immunoglobulin lambda light-chain restriction, indicating PBL. Furthermore, gastric differentiated adenocarcinoma was incidentally detected in some biopsy samples. Finally, a total gastrectomy was performed, and the postoperative course was uneventful. The patient is currently alive, 15 months after the initial diagnosis. This case reveals an endoscopic feature of gastric PBL and suggests the rare possibility that gastric PBL may coexist with adenocarcinoma.

**Supplementary Information:**

The online version contains supplementary material available at 10.1007/s13691-025-00751-4.

## Introduction

Plasmablastic lymphoma (PBL) is a rare lymphoma with a poor prognosis. It is classified as a large B-cell lymphoma within the category of mature B-cell neoplasms in the 5th edition of the World Health Organization Classification [1]. PBL was initially described in 1997 as a lymphoma with a predilection for the oral cavity in human immunodeficiency virus (HIV)-positive individuals [2]. Subsequently, PBL has been recognized as an aggressive lymphoma occurring in the setting of HIV, Epstein-Barr virus (EBV), or immunodeficiency [3–5]. Scattered reports have documented PBL occurrences outside the oral cavity, including in the gastrointestinal tract, lymph nodes, and skin in immunocompetent individuals [6]. Of the few reported cases of PBL in the gastrointestinal tract [7, 8], those occurring in the colorectum are more common than those occurring in the stomach. In addition, the endoscopic findings of gastric PBL are not well known. Here, we describe a case of primary gastric PBL with multiple polypoid lesions in an HIV- and EBV-negative patient. The patient underwent surgical resection after the coexistence of gastric cancer was diagnosed via endoscopic biopsy during chemotherapy.

### Case report

**Fig. 1:**
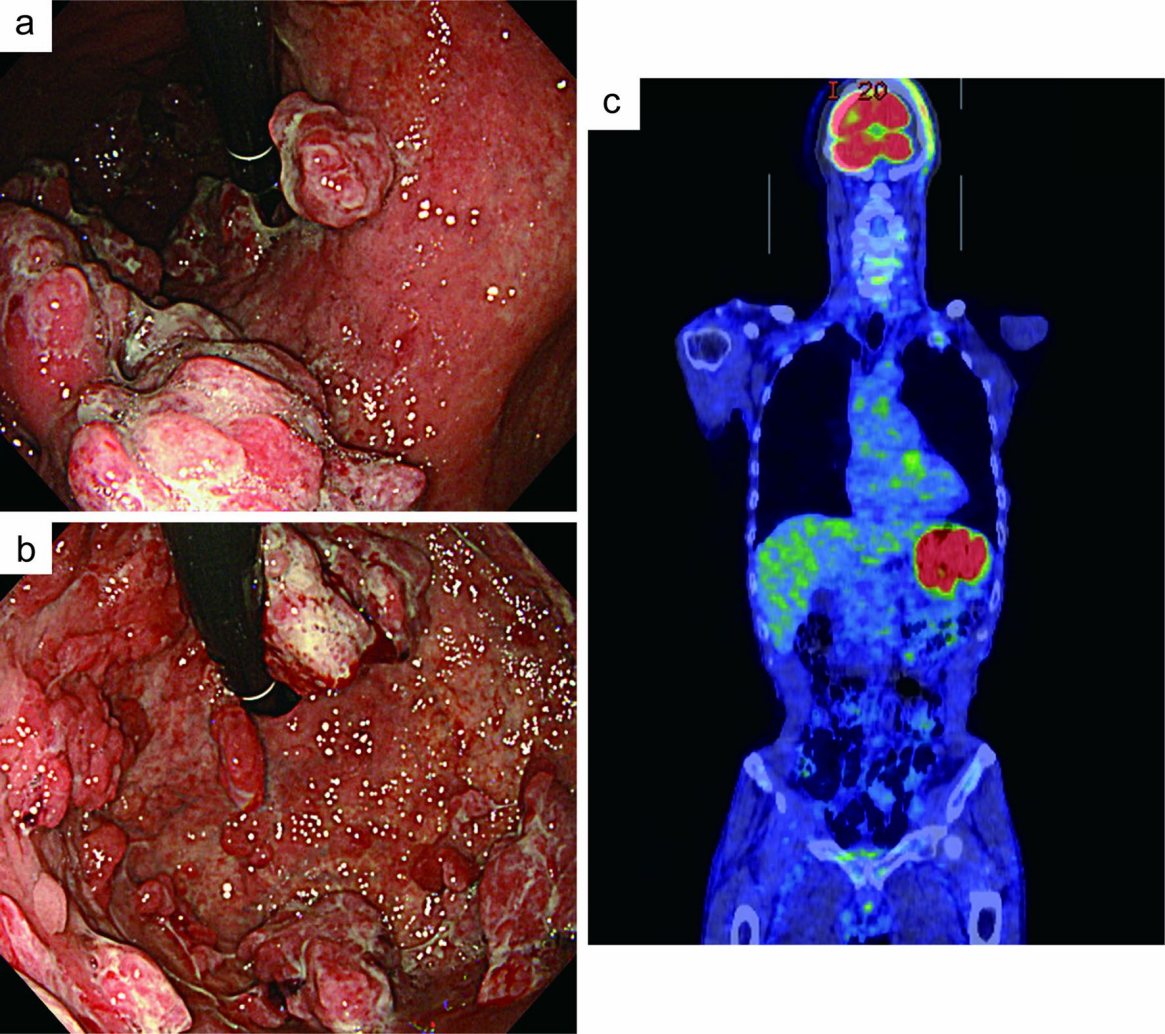
Endoscopic images and positron emission tomography/computed tomography images on initial examination. **a** Esophagogastroduodenoscopy showed multiple polypoid tumors in the body of the stomach. **b** Endoscopic image of the fundus of the stomach. **c** Positron emission tomography/computed tomography showed localized accumulation in the stomach

**Fig. 2:**
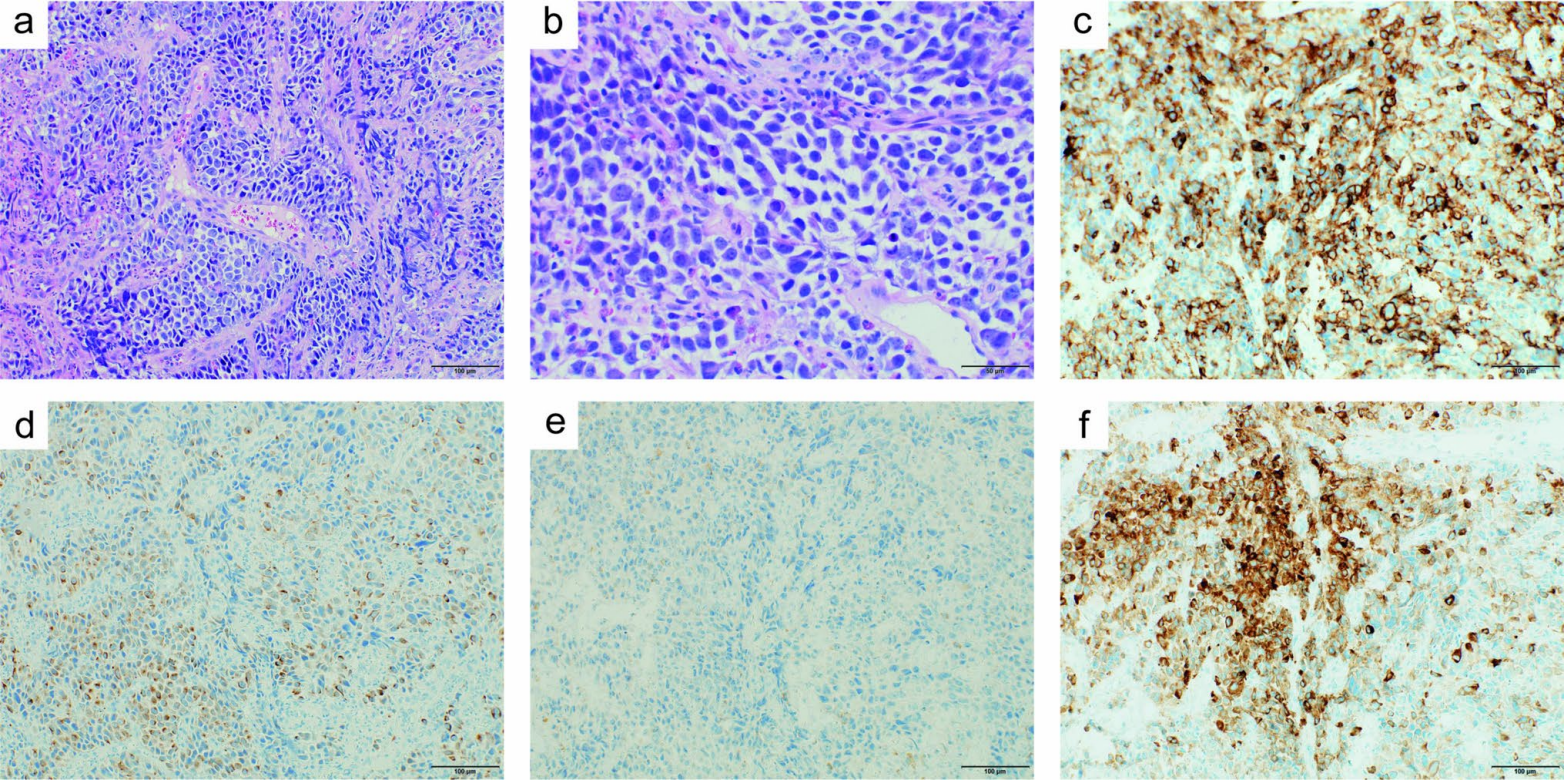
Histologic and immunohistochemical analysis of the first biopsy specimen. **a**, **b** Hematoxylin–Eosin staining showed infiltration of atypical tumor cells in the mucosa. **c** Tumor cells are positive for leukocyte common antigen. **d** Tumor cells are weakly positive for AE1/AE3. **e** Tumor cells are focally positive for CD20. **f** Tumor cells are positive for CD79a. Magnification 200 × (**a, c–f**) and 400 × (**b**)

A 72-year-old man presented with upper abdominal discomfort that had persisted for a month. The patient had no specific medical or family history. A physical examination revealed no peripheral lymphadenopathy or hepatosplenomegaly. The patient had mild upper abdominal tenderness. Laboratory data showed a white blood cell count of 11,700/μL, hemoglobin concentrations of 12.3 g/dL, platelet count of 491,000/µL, total protein of 5.6 g/dL, albumin of 2.4 g/dL, serum lactate dehydrogenase of 173 U/L, blood urea nitrogen of 15.1 mg/dL, creatinine of 0.73 mg/dL, serum calcium of 8.5 mg/dL, C-reactive protein of 6.24 mg/dL, and soluble interleukin-2 receptor of 583 U/mL. Monoclonal serum or urine immunoglobulins were not detected. The serum carcinoembryonic antigen and carbohydrate antigen 19-9 were normal. HIV, hepatitis B virus, and hepatitis C virus serological tests were all negative. The serum anti-*Helicobacter pylori* IgG antibody titer was 15 U/mL. Esophagogastroduodenoscopy (EGD) showed multiple raised tumors of variable sizes in the fundus and body of the stomach (Fig. 1a, b). The uneven tumor surfaces were white-coated, and the surface structures were difficult to observe. Contrast-enhanced computed tomography (CT) showed thickening of the stomach wall, and positron emission tomography (PET)/CT showed localized accumulation at the same site (Fig. 1c). Metastases to the lymph nodes or other organs were not observed. Pathohistological analysis of multiple biopsies from the gastric tumors showed diffuse proliferation of large tumor cells with a high N/C ratio and strong atypia, suggesting lymphoma or undifferentiated carcinoma (Fig. 2a, b). Immunohistochemical (IHC) analysis showed that the tumor cells were positive for leukocyte common antigen (Fig. 2c), CD79a (Fig. 2f), BCL2, and MUM1, weakly and focally positive for AE1/AE3 (Fig. 2d), CAM5.2, CD20 (Fig. 2e), CD30, and BCL6, and negative for CD3, CD5, and CD10. The expression of c-MYC was positive in 80% of the tumor cells, and the Ki67 labeling index was approximately 100%. EBV-encoded RNA in situ hybridization (EBER-ISH) was negative. On histopathological assessment, diffuse large B-cell lymphoma (DLBCL) was considered the most likely diagnosis. No abnormal lymphocytes were found on the iliac bone marrow examination. Hence, the disease was classified as stage I in the Lugano classification.

**Fig. 3:**
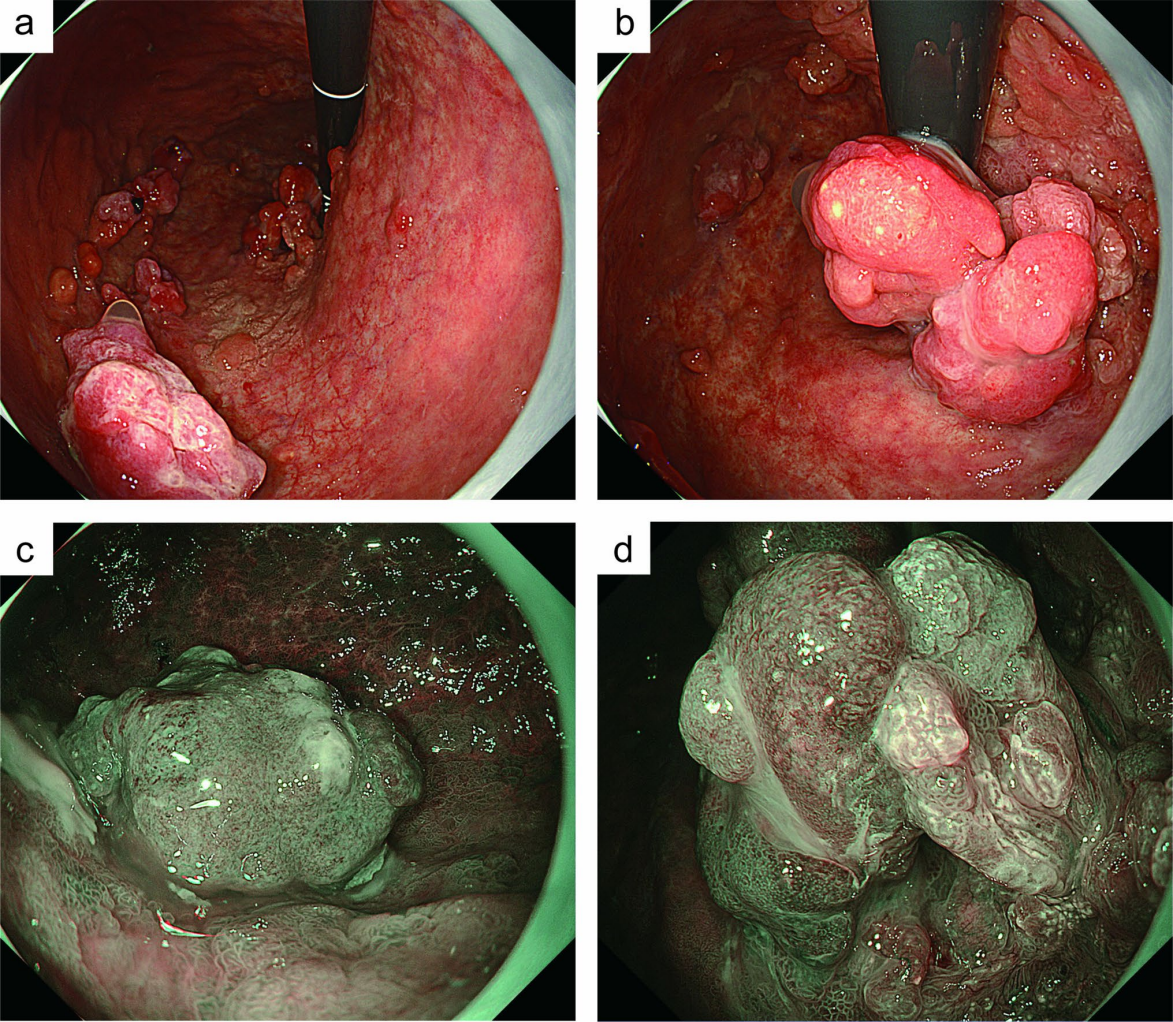
Endoscopic images after six cycles of chemotherapy. **a** Esophagogastroduodenoscopy shows a partial reduction of the tumor in the body of the stomach. **b** Endoscopic image of the fundus of the stomach. **c** Narrow-band image of the tumor in the body of stomach. **d** Magnified narrow-band image of the tumor in the fundus of the stomach

**Fig. 4:**
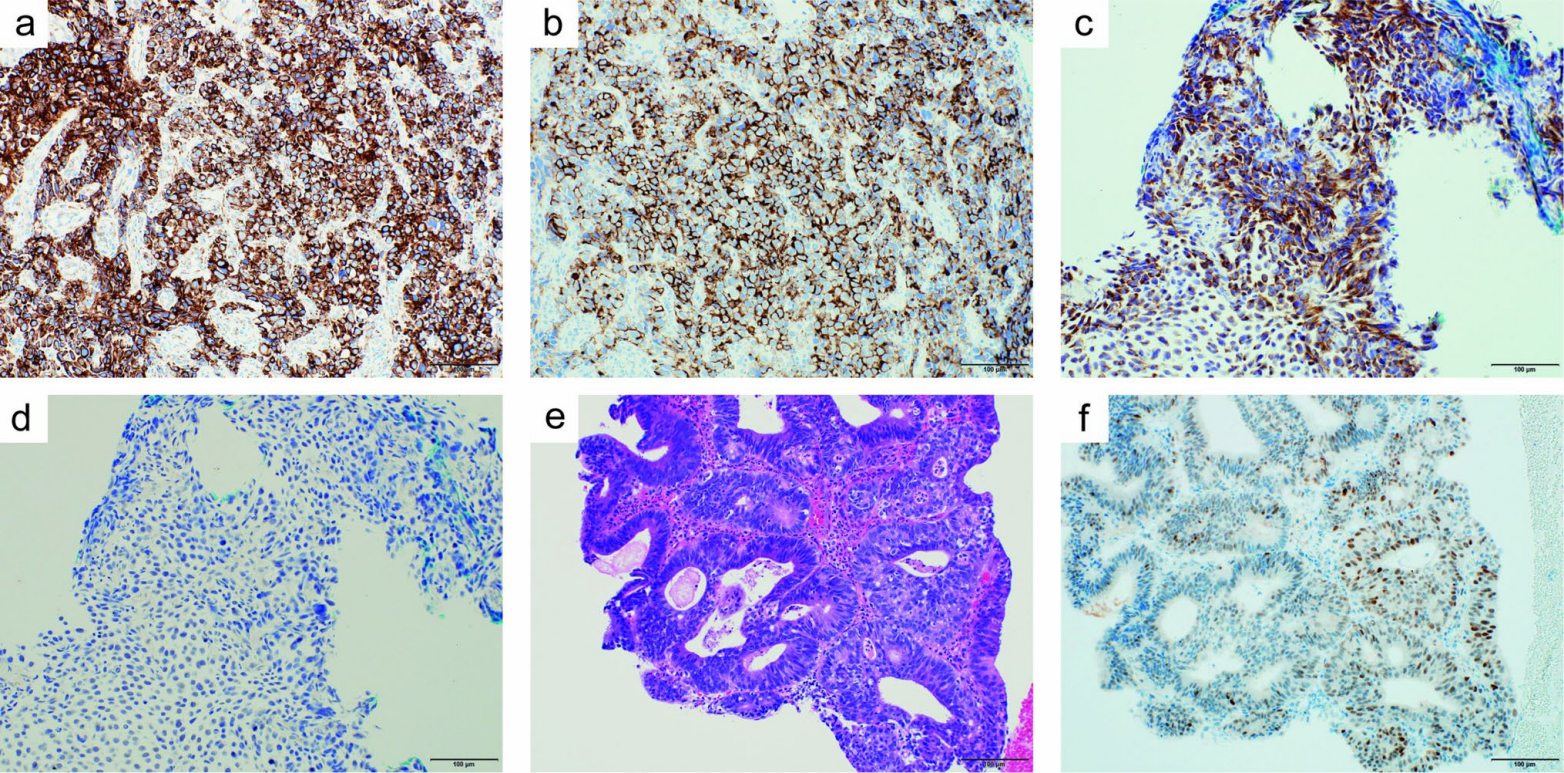
Histologic and immunohistochemical analysis of second biopsy specimen. **a** Tumor cells are strongly positive for CD38. **b** Tumor cells are strongly positive for CD138. **c** Immunostaining is positive for kappa light chain. **d** Immunostaining is negative for lambda light chain. **e** Hematoxylin–Eosin stains show well-differentiated adenocarcinoma in the epithelium. **f** Immunostaining of p53 protein on adenocarcinoma cells. Magnification 200 ×

**Fig. 5:**
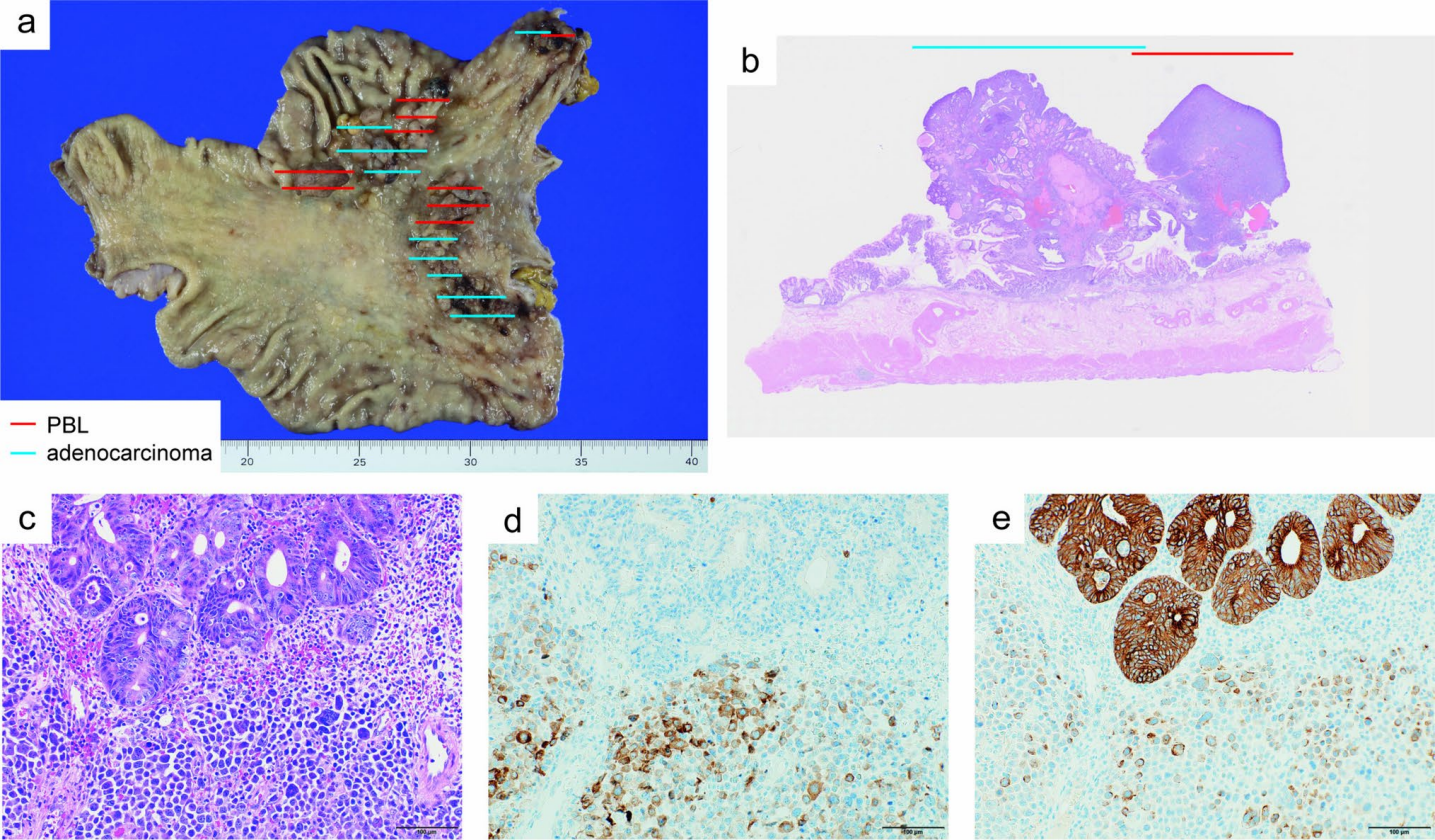
Macroscopic and microscopic findings of a surgical specimen. **a** Numerous small and large raised lesions are observed in the fundus and body of the stomach. **b** Low-magnification image of a sample with coexisting plasmablastic lymphoma (PBL) and adenocarcinoma. **c** High-magnification image of a sample with coexisting PBL and adenocarcinoma. **d** PBL cells are positive for CD79a. **e** Gastric adenocarcinoma cells are strongly positive for AE1/AE3. Magnification 20 × (**b**) and 200 × (**c**–**e**)

The patient was administered rituximab, cyclophosphamide, doxorubicin, vincristine, and prednisone (R-CHOP) chemotherapy. After six courses of R-CHOP chemotherapy, EGD showed a partial reduction in the stomach tumor (Fig. 3a, b). Narrow-band imaging revealed solid tumors with no visible glandular structures on the surface (Fig. 3c), and tumors with irregular villous or papillary surface structures (Fig. 3d). These findings differed from the typical endoscopic findings of gastric DLBCL, prompting the repetition of residual tumor biopsies. Because immunostaining of tumor cells for CD20 was not characteristic of DLBCL, histopathology was re-examined, and IHC analysis revealed that the tumor cells were strongly positive for CD38 and CD138 (Fig. 4a, b). In addition, the cells showed cytoplasm immunoglobulin lambda light chain restriction, suggesting PBL based on these findings (Fig. 4c, d). Furthermore, gastric differentiated adenocarcinoma was incidentally detected in some biopsy samples (Fig. 4e, f). The final diagnosis was the coexistence of primary gastric PBL and gastric adenocarcinoma. A CT scan performed after six courses of R-CHOP chemotherapy also showed that the tumor lesion was confined to the stomach, therefore, the patient underwent total gastrectomy with lymph-node dissection. Histologic images of the excised specimens are shown in Fig. 5. Macroscopic examination revealed multiple raised lesions in the upper and middle portions of the stomach (Fig. 5a). Microscopic findings showed that some raised lesions coexisted with PBL and gastric adenocarcinoma (Fig. 5b–e). PBL and adenocarcinoma developed within the epithelium, with no evidence of submucosal invasion or lymph-node metastasis. The histologic type of gastric cancer was a well-differentiated tubular adenocarcinoma, localized to the mucosal layer, with no lymphovascular infiltration and HER-2 negative. The postoperative course was favorable, and the patient is on a treatment-free follow-up. The patient is currently alive 15 months after the initial diagnosis.

## Discussion

This case highlights two important clinical issues. First, gastric PBL can present as multiple polypoid lesions in HIV- and EBV-negative patients. Second, gastric PBL may coexist with gastric adenocarcinoma. To the best of our knowledge, this is the first case presenting endoscopic images of multiple polypoid lesions of gastric PBL and reporting the coexistence of PBL with gastric adenocarcinoma.

First, gastric PBL can exhibit multiple polypoid lesions in HIV- and EBV-negative patients. Although PBL of the stomach was first reported in 1998 [9], only a few reports have been published. To our knowledge, 11 cases of gastric PBL were reported from 1998–2023 [9–19]. The clinical and imaging characteristics of the published cases are summarized in Table 1. Seven of the 11 reports described endoscopic findings, and three of these included endoscopic images. Four of the six cases showed ulcerative lesions, while threes exhibited polypoid masses or irregularly raised lesions; however, no endoscopic images were available. This is the first report of endoscopic imaging of multiple polypoid lesions in gastric PBL.

**Table 1:** A summary of the reported cases of gastric plasmablastic lymphoma

	Author	Year	Age/ sex	HIV/ EBV	Symptom	Tumor location	Endoscopic finding	Treatment	Outcome
1	Pruneri et al. [9]	1998	53/F	−/NR	Epigastric distension	NR	Large polypoid mass (no image)	Surgery ProMACE-cytaBOM	Alive at 19 mo
2	Hashimoto et al. [10]	2012	70/F	−/−	Melena, loss of appetite	NR	NL	Operation CHOP DeVIC	DOD during second cycles of DeVIC
3	Chapman-Fredricks et al. [11]	2012	46/F	+ / +	Nausea, melena, diarrhea, etc	Fundus	NL	EPOCH	NR
4	Mihaljevic et al. [12]	2012	60/M	−/−	Abdominal pain, melena	Entire stomach	NL	CHOP	DOD after one cycle of CHOP
5	Marques et la [13]	2013	82/F	−/NR	Melena, epigastric pain, weight loss, etc	Fundus, body, antrum	Ulceration, mucosal hyperemia and pallor	CHOP	DOD after one cycle of CHOP
6	Huang et al. [14]	2015	21/M	−/−	Abdominal fullness, diarrhea	NR	Large polypoid mass (no image)	None	DOD after diagnosis
7	Amenta et al. [15]	2016	82/M	−/ +	NR	Cardia, fundus	NL	CHOP Radiation	DOD 11 mo after diagnosis
8	Komaranchath et al. [16]	2016	20/M	−/−	Epigastric pain, vomiting	Antrum	Irregular raised lesion (no image)	CHOP	PD after six cycles of CHOP
9	Angeleri et al. [17]	2016	50/M	+ / +	Fatigue, weakness, weight loss, etc	Fundus	Enlarged folds, ulceration (no image)	NR	NR
10	Johan et al. [18]	2021	74/M	−/−	Shortness of breath, abdominal pain	Fundus, body	Multiple dish-like ulcers	None	DOD before treatment
11	Pereira et al. [19]	2023	50/M	+ / +	Anemia	Fundus, body	Mucosal thickening, ulceration	None	DOD at 10 days
12	Our case	2025	72/M	−/−	Abdominal discomfort	Fundus, body	Multiple polypoid tumors	R-CHOP, Surgery	Alive at 15 mo

+: positive; -: negative; *NR* None reported; *NL* None listed; ProMACE-cytaBOM Prednisone, methotrexate, doxorubicin, cyclophosphamide, etoposide, cytarabine, bleomycin, vincristine, methotrexate; *CHOP* Cyclophosphamide, hydroxydaunorubicin, oncovin, prednisone; *R-CHOP* Rituximab, cyclophosphamide, hydroxydaunorubicin, oncovin, prednisone; *DeVIC* Dexamethasone, etoposide, ifosfamide, carboplatin; *EPOCH* Etoposide, vincristine, doxorubicin, cyclophosphamide, prednisolone; *DOD* Died of disease; *PD* Progressive disease

The endoscopic findings of gastric lymphoma vary. Primary gastric lymphoma is mostly a B-cell non-Hodgkin’s lymphoma, with mucosa-associated lymphoid tissue (MALT) lymphoma and DLBCL [20]. The endoscopic appearance in MALT lymphoma tends to appear as a superficial type, whereas gastric DLBCL is a mass/polypoid or ulcerative type, and these lesions are often multiple [21, 22]. The endoscopic findings of gastric PBL may be similar to those of DLBCL, as previous reports have shown ulceration and raised/polypoid lesions. Notably, gastric PBL appears to exhibit a predilection for the upper to middle part of the stomach, with 8 out of 12 reported cases, including the present case (three cases did not report the location), showcasing lesions in the fundus and body of the stomach (Table 1). In addition to these findings, further cases are needed to clarify the endoscopic features of gastric PBL.

HIV and EBV have been implicated in the development of PBL [2, 3]. However, they are not necessarily involved in PBL of the gastrointestinal tract. In a study of 590 PBL cases, 43 out of 83 PBL cases (52%) occurring in the gastrointestinal tract were HIV-positive [6]. In a study of PBL in the intestinal tract, 6 of 17 cases (27%) were HIV-positive, and 9 of 16 cases (56%) were EBV-positive [23]. In a case series of gastrointestinal PBL in HIV-negative cases, 19 of 31 (61%) cases were EBV-positive [7]. Regarding gastric PBL, 3 of 11 (27%) cases were HIV-positive and four of nine (44%; two cases did not report EBV status) were EBV-positive (Table 1). Both HIV and EBV tested negative in this case, and their involvement in the development of gastric PBL may not have been significant. This suggests the need to include PBL in the differential diagnosis of gastrointestinal tract lymphomas, even in patients without HIV or EBV infection.

PBL is sometimes difficult to diagnose pathologically due to its rarity, especially when it occurs in the extraoral region of HIV- or EBV-negative patients. PBLs mainly have cytomorphological features, such as large plasma cells or large immunoblasts expressing plasma cell markers and lacking B-cell markers [6, 24]. Differential diagnoses of PBL include plasmablastic myeloma, DLBCL (not otherwise specified), and undifferentiated carcinoma. In this case, plasmablastic myeloma was excluded because of the absence of features favoring myeloma, such as monoclonal paraproteinemia and hypercalcemia. In addition, the differentiation between DLBCL and undifferentiated carcinoma from PBL typically relies on the expression of pan-B-cell markers and cytokeratin, respectively. This patient had an unusual phenotype with partial expression of B-cell marker (CD20) and cytokeratins (AE1/AE3 and CAM5.2), which led to difficulties in the initial diagnosis [25]. Furthermore, the combination of CD20 and PAX5 expressions is useful in classifying DLBCL and PBL, with negative or weakly positive CD20 and PAX5 being characteristic phenotypes of PBL [26]. In this case, subsequent analysis revealed negative PAX5 expression in the tumor cells (Supplementary Fig. 1), combined with weak CD20 expression, indicating the diagnosis of PBL.

The second clinical suggestion is that gastric PBL can coexist with gastric adenocarcinoma. Gastric lymphoma and gastric cancer are rarely diagnosed simultaneously. A previous Japanese study reported that 4 of 121 (3.3%) primary gastric lymphomas resected surgically coexisted with gastric adenocarcinoma [27]. In a multicenter study in China, 5% (24/474) of patients with gastric lymphoma were diagnosed with gastric cancer, and only one of these patients was synchronously diagnosed with lymphoma and gastric cancer [28]. Most of the lymphomas in these studies were DLBCL or MALT lymphomas. Given the rarity of gastric PBL, reports on its complication with gastric cancer are absent. Differentiating gastric PBL from other lymphomas and diagnosing synchronous gastric cancer based on endoscopic appearance can be difficult. In this case, the second biopsy revealed gastric PBL, and the coexistence of gastric cancer was confirmed. Repeated pathological examinations contribute to the accurate diagnosis of rare lymphomas and the detection of synchronous gastric cancer.

The primary etiology of gastric adenocarcinoma and MALT lymphoma is *Helicobacter pylori* (*H. pylori*) infection [29, 30]. Moreover, *H. pylori* has also been implicated in some gastric DLBCL [31]. Precancerous conditions resulting from chronic gastritis caused by *H. pylori* contribute to the synchronous or metachronous occurrence of gastric cancer and lymphoma [28]. However, the role of *H. pylori* infection in gastric PBL development remains unclear. Previous studies have identified HIV [2], EBV [6], organ transplantation [5], chronic inflammatory disease [32], and immunosenescence in the elderly [5, 33] as factors associated with PBL. In our case, age was the sole relevant factor, with no history of immunodeficiency or immunosuppressive treatment. In this case, the patient had positive serological test results for *H. pylori*. As with other gastric lymphomas, the association between gastric PBL and *H. pylori* should be investigated in future studies. Furthermore, EBER-ISH was negative in PBL cells but positive in gastric adenocarcinoma in this case (Supplementary Fig. 2). EBV-associated gastric cancer is characterized by undifferentiated adenocarcinoma with lymphocyte infiltration in the stroma, and a relatively low frequency of lymph-node metastasis has been reported [34, 35]. The present case did not have the characteristic histology of EBV-associated gastric cancer, and the clinical significance of EBER positivity in intramucosal differentiated adenocarcinoma is unknown; however, the etiology of PBL and gastric adenocarcinoma in this case was suggested to be different.

The treatment for gastric PBL has not yet been established. In previous reports, a relatively high proportion of patients were treated with systemic chemotherapy, such as CHOP, because of the advanced stage of the disease; however, the prognosis was poor. Surgical resection was performed in two patients, both with postoperative chemotherapy (ProMACE-cytaBOM and CHOP), and one patient survived 19 months after diagnosis (Table 1). In this case, the gastric PBL was confined to the stomach, and the gastric cancer was an intramucosal adenocarcinoma without external metastasis; therefore, surgical gastrectomy seemed appropriate. Chemotherapy regimens in the postoperative period and relapse should be discussed in future.

In conclusion, our case revealed some endoscopic features of gastric PBL and suggested the rare possibility that gastric PBL may coexist with adenocarcinoma. Repeated pathologic examinations contribute to the accurate diagnosis of rare lymphomas and the detection of synchronous gastric cancer.

## Supplementary Information

Below is the link to the electronic supplementary material.

Supplementary file1 (PDF 399 KB)

## Data Availability

Data and materials are available upon request to the corresponding author.
