# Supplementary material for: Coexistence of plasmablastic lymphoma and adenocarcinoma in the stomach: a case report and literature review
Source: Int Cancer Conf J. 2025 Feb 15;14(2):155–62. doi: 10.1007/s13691-025-00751-4 (PMC11950613; doi:10.1007/s13691-025-00751-4)
Supplement: Supplementary file 1 — Supplementary file1 (PDF 399 KB) [file 13691_2025_751_MOESM1_ESM.pdf]

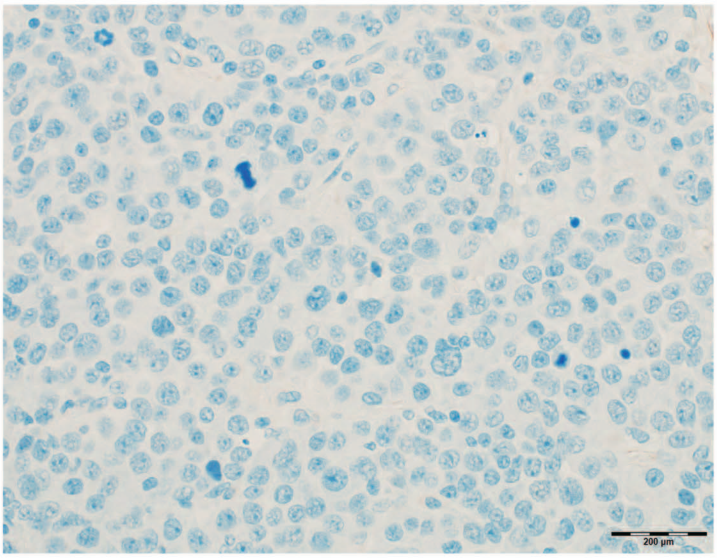

Supplementary Fig. 1 Immunostaining for PAX5 in tumor cells

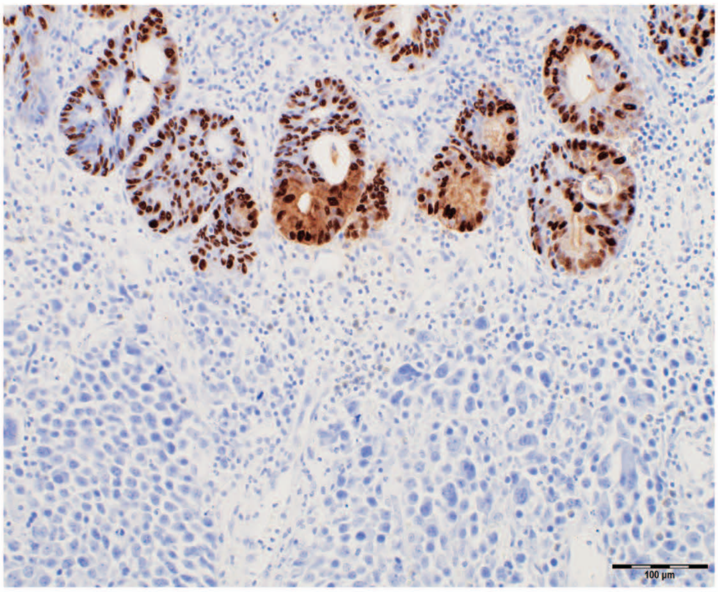

Supplementary Fig.2 EBER expression of plasmablastic lymphoma
and gastric adenocarcinoma.
